# Methods to Investigate the Deformability of RBC During Malaria

**DOI:** 10.3389/fphys.2019.01613

**Published:** 2020-01-21

**Authors:** Mallorie Depond, Benoit Henry, Pierre Buffet, Papa Alioune Ndour

**Affiliations:** ^1^UMR_S1134, BIGR, Inserm, Universit de Paris, Paris, France; ^2^Institut National de la Transfusion Sanguine, Paris, France; ^3^Laboratory of Excellence GR-Ex, Paris, France

**Keywords:** deformability, erythrocytes, *Plasmodium*, malaria, ektacytometry, microfluidics, microsphiltration, micropipette

## Abstract

Despite a 30% decline in mortality since 2000, malaria still affected 219 million subjects and caused 435,000 deaths in 2017. Red blood cells (RBC) host *Plasmodium* parasites that cause malaria, of which *Plasmodium falciparum* is the most pathogenic. The deformability of RBC is markedly modified by invasion and development of *P. falciparum*. Surface membrane area is potentially impacted by parasite entry and development, the cytoskeleton is modified by parasite proteins and cytosol viscosity is altered by parasite metabolism. RBC hosting mature parasites (second half of the asexual erythrocytic cycle) are abnormally stiff but the main reason for their absence from the circulation is their adherence to endothelial cells, mediated by parasite proteins exposed at the infected-RBC surface. By contrast, the circulation of non-adherent rings and gametocytes, depends predominantly on deformability. Altered deformability of rings and of uninfected-RBC altered by malaria infection is an important determinant of malaria pathogenesis. It also impacts the response to antimalarial therapy. Unlike conventional antimalarials that target mature stages, currently recommended first-line artemisinin derivatives and the emerging spiroindolones act on circulating rings. Methods to investigate the deformability of RBC are therefore critical to understand the clearance of infected- and uninfected-RBC in malaria. Herein, we review the main methods to assess the deformability of *P. falciparum* infected-RBC, and their contribution to the understanding of how *P. falciparum* infection causes disease, how the parasite is transmitted and how antimalarial drugs induce parasite clearance.

## Introduction

Red blood cells (RBC) are essential for oxygen delivery to organs and thus must circulate in narrow blood vessels without being destroyed. These cells, devoid of nucleus and organelles, have unique properties of deformability, i.e., exquisite ability to shape modification upon mechanical constraints. This enables their circulation in blood capillaries, which are narrower than the RBC main diameter. The biconcave shape of RBC increases their surface-to-volume ratio (An and Mohandas, 2008). The deformability of RBC depends on three parameters: (i) the membrane elasticity that is mainly dependent on cytoskeletal components, (ii) the cytoplasmic viscosity that depends on intracellular ion and hemoglobin concentration/state, and (iii) the surface-to-volume ratio. The balance among these three parameters can be altered during malaria (Lavazec, 2017).

**FIGURE 1:**
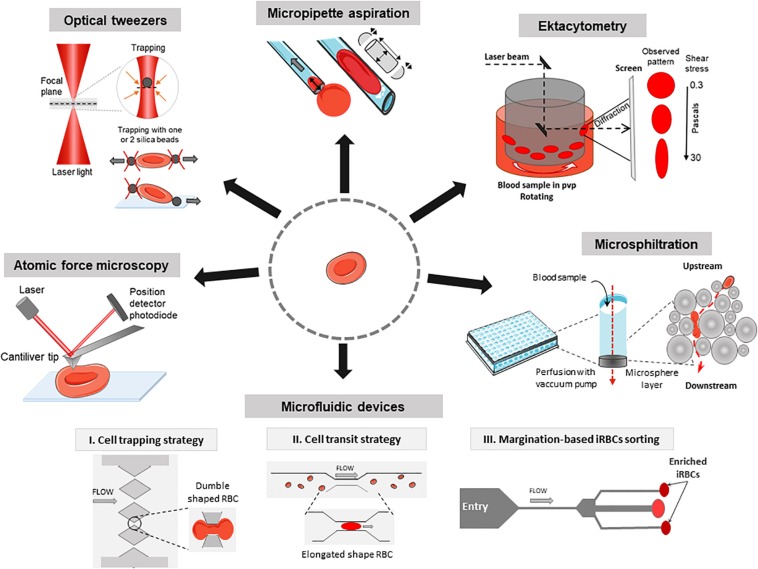
Methods to investigate the deformability of RBC during malaria. *Atomic force microscopy*: Imaging mode reconstructs a 3D topography of the RBC surface using a cantiliver tip that scans in *x* and *y* directions. Force spectroscopy mode measures forces in *z* direction and thus gives information about local strength, elasticity, and stiffness of single RBC. *Optical tweezers:* A focused laser beam provides a three-dimensional gradient to apply very small forces and manipulate silica beads that adhere electrostatically to a single RBC. *Micropipette aspiration:* The surface of a single RBC is aspirated into the mouth of a glass pipette while suction pressures are applied. When the RBC is partially aspirated, the length of the tongue informs on membrane deformability in the corresponding local area. When the whole RBC is aspirated, the volume and the surface can be computed considering the RBC as two hemispheres separated by a cylinder. *Ektacytometry:* Diffraction pattern of a laser beam through a population of RBC diluted in a viscous medium (PVP) and submitted to a shear flow from 0.3 to 30 Pa, provides the elongation index EI. *Microsphiltration:* Assesses the ability of RBC to squeeze through narrow slits between metallic microspheres under flow, mimicking splenic filtration. The retained fraction is computed from upstream and downstream concentrations of the population of interest. *Microfluidics:* The control of fluid flow is via micropumps/microvalves then the behavior of single RBC is tracked by videomicroscopy on a specifically designed polydimethylsiloxane (a silicon-based organic polymer, PDMS) biochip. *I, Cell trapping strategy* (based on Gambhire et al., 2017): Time of transit and rate of RBC sequestration in submicrometers channels, biomimetic splenic slits are quantified. *II, Cell transit strategy* (based on Antia et al., 2007): rheological responses to changing dimensions of capillaries with shapes and sizes similar to small blood vessels are observed. *III, Margination-based iRBCs sorting* (based on Hou et al., 2010): cell margination is mimicked for the separation of iRBCs from whole blood based on their reduced deformability.

Despite the decline in malaria-specific mortality, there were 435,000 deaths in 2017 (WHO report 2017), most attributable to *Plasmodium falciparum*. *Plasmodia* are protozoan parasites that cause malaria. During the intra-erythrocytic stage of infection, RBC undergo marked changes. Upon parasite internalization (invasion), RBC undergo a very transient shape change (echinocytosis) before recovering a normal biconcave shape (Gilson and Crabb, 2009). During the parasite asexual replication (including the sequential ring, trophozoite, and schizont stages) and sexual development (female and male gametocytes stage I–V), parasite maturation induces changes in the host RBC with novel proteins synthesis (Gilson et al., 2017; Ndour et al., 2017; Weißbach et al., 2017). As the parasite develops, the infected RBC (iRBC) loses its biconcave shape and progressively becomes spherical and rigid (Cranston et al., 1984), and its surface area-to-volume ratio decreases. The loss of RBC deformability is not limited to mature stages but starts soon after parasite invasion. During the ring stage (i.e., within the first 16–24 h after RBC invasion by the parasite), iRBC undergo up to 9.6% surface area loss (Safeukui et al., 2013; Jaureguiberry et al., 2014). More than 50% of ring-iRBC are retained upon *ex vivo* transfusion through human spleens (Safeukui et al., 2008, 2013; Deplaine et al., 2011) and have been recently shown to accumulate by several orders of magnitude in the spleen of asymptomatic carriers undergoing splenectomy for trauma in Indonesian Papua (Kho, 2019). These retention and accumulation processes stem from the human spleen physiological function to control the RBC deformability. RBC navigating through the splenic red pulp must indeed squeeze through small intercellular slits in the wall of venous sinuses (Groom et al., 1991; Suwanarusk et al., 2004; Buffet et al., 2011). These splenic slits create a physical fitness test for RBC and for particles that they contain, which are cleared from the circulation if their geometry and deformability are altered (Safeukui et al., 2008; Pivkin et al., 2016; Li et al., 2018; Wojnarski et al., 2019). Retention of ring-infected and uninfected RBC, which are also partially altered during infection, are predicted to impact the pace of infection and to contribute to splenomegaly and anemia, two hallmarks of malaria in human subjects (Cranston et al., 1984; Buffet et al., 2009; Fernandez-Arias et al., 2016). Drug-induced alterations of the deformability of iRBC may also impact the efficacy of antimalarial regimens and the pace of treatment-induced parasite clearance. These observations on malaria pathogenesis and the deformability of RBC were generated through different methods. We review here these methods and their contribution to the understanding of how infection with *P. falciparum* causes disease, how the parasite is available for transmission to the Anopheles vector and how antimalarial drugs induce parasite clearance (see Table 1 and Figure 1).

**TABLE 1:** Literature overview of the main methods exploring the RBC deformability altered by malaria.

	**Outline of the method**	**Readout**	**Throughput/Limitations**	**References of *in vitro* studies**	**References of studies in human subjects**
Ektacytometry	Laser diffraction through a RBC population submitted to a shear flow from 0.3 to 30 Pa in a viscous medium	Elongation index EI, dimensionless value, from 0 (at low shear stress) to 0.65 (at high shear stress)	Low-medium, 10 min/sample. Population analysis.	Cranston et al., 1984; Maier et al., 2008	Dondorp et al., 1997, 1999; Dondorp et al., 2002; Ishioka et al., 2016; Barber et al., 2018
Micropipette aspiration	The surface of the cell is aspirated into the mouth of a glass pipette while suction pressures are applied	Under microscope, the leading edge of the membrane surface is tracked with an accuracy of ±25 nm and enables the quantification of the membrane shear elastic modulus	Low, single cell, requires training manipulator, no commercial source of micropipettes, precise but time consuming	Nash et al., 1989; Glenister et al., 2002; Aingaran et al., 2012; Tiburcio et al., 2012; Shojaei-Baghini et al., 2013; Zhang et al., 2016	Nash et al., 1989; Barber et al., 2018
Microfluidics	Live observation of RBC navigating along narrow channels or across slits in specifically designed PDMS biochips. Controled flow via micropumps/microvalves	Ability of RBC to cross channels or slits, assessed by time of passage or sustained retention (quantitative). Shape deformation and shape recovery (qualitative or quantitative)	Low. Very informative but technically challenging. Qualitative and/or quantitative analysis	Shelby et al., 2003; Antia et al., 2008; Handayani et al., 2009; Herricks et al., 2009, 2012; Hou et al., 2010; Imai et al., 2010; Bow et al., 2011; Huang et al., 2013; Wu and Feng, 2013; Picot et al., 2015; Guo et al., 2016	Herricks et al., 2012
Microsphiltration	Measure of the ability of RBC to squeeze through narrow slits between metallic microspheres, mimicking splenic filtration	Retention or enrichment rates (RER%) by comparing upstream and downstream concentrations of the tested RBC subppulation	Medium (with single tip) to high (using 384-wells plate) RBC population analysis	Deplaine et al., 2011; Lavazec et al., 2012; Sanyal et al., 2012; Tiburcio et al., 2012; Duez et al., 2015; Ndour et al., 2015; Dearnley et al., 2016; Duez et al., 2018	Henry et al., 2018
Atomic Force Microscopy	*Imaging mode* reconstructs a 3-dimension topography of a RBC surface using a cantiliver tip that scans in *x* and *y* dimensions. *Force spectroscopy mode* measures forces in *z* direction and thus gives information about local strength, elasticity, and stiffness	Erythrocyte Young’s modulus is calculated from addition of multiple force curves, analyzed with a processing software	Low, single cell, requires training manipulator	Aikawa et al., 1996; Nagao et al., 2000; Li et al., 2006; Dearnley et al., 2016; Sisquella et al., 2017; Perez-Guaita et al., 2018 (AFM-IR)	Barber et al., 2018
Optical tweezers	Optical tweezers exert very small forces (picoNewtons) using a focused laser beam to manipulate dielectric particles	Forces in the picoNewton range are applied and displacements are measured in the nm range	Low, single cell, requires training manipulator	Mills et al., 2004, 2007; Suresh et al., 2005; Bambardekar et al., 2008; Fedosov et al., 2011, 2014; Hosseini and Feng, 2012; Ye et al., 2013	
Imaging flow cytometry	Combination of a flow cytometer with microscopy that takes pictures of focused cells	Each image results from the combination of sub-images with fluorescence emissions, scattered and transmitted light data. This process generates single-cell pictures that display sucellular fluorescent mapping	High	Safeukui et al., 2013; Jaureguiberry et al., 2014; Barber et al., 2018; Roussel et al., 2018	Barber et al., 2018

## Ektacytometry

The deformability of *P. falciparum*-infected RBC was first monitored by a rheoscope (counterrotating transparent cone-and-plate chamber to measure elongation of RBC under shear stress) which assesses the shape of individual RBC (Cranston et al., 1984). This method provided the first demonstration of a reduced deformability of iRBC which may induce their retention in the spleen. Ektacytometry uses a Laser-assisted Optical Rotational Red Cell Analyzer (LORRCA, RR Mechatronics, Netherlands) to measure the diffraction pattern of sheared RBC resuspended in a viscous medium at 37°C to determine their elongation index (EI). It rapidly measures the average deformability of an RBC population (Figure 1). Ektacytometry has been widely used to assess RBC deformability in pathologic conditions, both in inheritable RBC disorders and malaria (Dondorp et al., 1999; Da Costa et al., 2016). EI positively correlated with a greater RBC deformability (Baskurt et al., 2009). Shear stresses of 1.7 Pa are encountered in capillaries. Shear stresses of 30 Pa provide information on the RBC surface-to-volume ratio and may be a proxy for the ability of RBC to cross splenic slits (Barber et al., 2018). Reduced EI of circulating RBC, which contain a vast majority of uninfected RBC, in patients with malaria correlates with disease severity (Dondorp et al., 1999, 2002; Ishioka et al., 2016; Barber et al., 2018). A large-scale gene knockout strategy was combined with ektacytometry to determine the role of parasitic exported proteins in stiffness (Maier et al., 2008). This study revealed eight genes encoding proteins for export of the parasite adhesin PfEMP1 and some playing a role in increasing rigidity of iRBC. RBC population containing stiffened cells gives a diamond-shaped diffraction pattern instead of an elliptical-shaped diffraction pattern. Recent works based on mathematical models have proposed new methods to quantify this fraction of poorly deformable RBC in clinical samples (Streekstra et al., 2010; Parrow et al., 2018). Compared to the LORRCA which allows populational analysis only, the recent Automated-Rheoscope-and-Cell-Analyzer (ARCA) analyzes single cell deformability thanks to the advanced image analysis and can determine the RBC deformability distribution (Dobbe et al., 2002).

## Micropipette Aspiration

The mechanical properties of RBC can be studied through micropipette aspiration, firstly described as a “cell elastimeter,” in which the surface of the cell is aspirated into the mouth of a glass pipette (Havell et al., 1978). The cell tongue length is quantified with an accuracy of ±25 nm. The cell undergoes suction pressures, from 0.1 pN/μm^2^ to almost atmospheric pressure, and traction forces, from about 10 pN to 10^4^nN (Hochmuth, 2000). The key output is the membrane shear modulus (i.e., membrane extension, in pN/μm). Micropipette aspiration requires training and it is a demanding single cell method (Table 1). RBC infected with rings and mature stages require 1.5 and 4–6 times more pressure than normal RBC to enter into 3 μm-wide pipettes, respectively (Nash et al., 1989). Micropipette aspiration assays have shown that the parasite proteins KAHRP and PfEMP3 contribute to membrane rigidification of mature-iRBC (Glenister et al., 2002). This same technique showed that that immature gametocytes display a decrease in membrane deformability as compared to uninfected or ring-iRBC. However, gametocyte deformability is fully restored during the transition from the stage IV to the final stage V (mature gametocytes). Along with observations made with microsphiltration (Tiburcio et al., 2012; see below), these observations shed light on how gametocytes are able to circulate for transmission, while immatures do not (Aingaran et al., 2012). Micropipette aspiration showed that ring-stage iRBC exposed to KAE609/NITD609/Cipargamin, a novel potent antimalarial drug, becomes spherical and rigid (Zhang et al., 2016), likely explaining why this drug induces the fastest parasite clearance ever observed in humans (Rottmann et al., 2010; White et al., 2014). New automated micropipette aspiration has been launched with lower operator skill requirements and faster sample processing. Using vision-based and position controls, the system monitors a micromanipulator, a motorized translation stage, and a custom-built pressure system to position a micropipette to approach a cell, form a seal, and aspirate the cell into the micropipette (Shojaei-Baghini et al., 2013).

## Microfluidics

Microfluidic devices coupled to videomicroscopy are powerful tools to explore how RBC behave in capillaries or splenic slits, in physiology and disease, including malaria (Shelby et al., 2003; Rigat-Brugarolas et al., 2014; Picot et al., 2015; Guo et al., 2016). Observation using microfluidic devices have been combined with *in silico* simulations to predict RBC deformability (Bow et al., 2011). RBC retention rates or time of RBC transit through the device are the main readouts (Table 1 and Figure 1; Antia et al., 2008; Kang and Lee, 2018). The circulatory spaces within the chips can be designed to mimic capillaries or splenic slits with homogeneous sizes (Gambhire et al., 2017) or to enter into channels/slits with decreasing width (Herricks et al., 2009; Picot et al., 2015). While normal RBC easily cross 2 μm-wide slits, schizont-iRBC are generally blocked in 2–5 μm constrictions. Wedge-shaped microfluidic channels were used to evaluate minimum cylindrical diameter of RBC, which differ between normal RBC and iRBC (Herricks et al., 2009, 2012). Microfluidics showed the sharp contrast between rigid *P. falciparum*-iRBC and deformable *Plasmodium vivax*-iRBC at their respective mature stage. which may explain why a proportion of circulating *P. vivax-*iRBC escape splenic clearance (Handayani et al., 2009). Deformation-based cell margination is the displacement of less deformable cells toward a blood vessel wall narrower than 300 μm. Stiffer iRBC behave like leukocytes and undergo margination in a microfluidic model (Hou et al., 2010). This enables a highly efficient separation and collection of iRBC from whole blood without label or dye, from 75% for early ring stage iRBC to >90% for late trophozoite/schizonts. Microfluidics are thus powerful tools in the field of malaria drug discovery based on deformability. Recent comprehensive reviews have described microfluidic platforms to measure RBC deformability (Bento et al., 2018; Kang and Lee, 2018). Using a particle-based model, some simulations of microfluidic channels occlusion provide hemodynamic parameters of transit of iRBC (Imai et al., 2010; Wu and Feng, 2013).

## Microsphiltration

Microsphiltration has been designed to mimic the mechanical sensing of RBC as they cross inter-endothelial slits in the human spleen (Figure 1). Calibrated metal microspheres 5–25 μm in diameter, shape a matrix that assesses the deformability of iRBC mixed with normal RBC (Figure 1). The upstream and downstream proportions of iRBC (i.e., parasitemia quantified either on Giemsa-stained smears or by flow cytometry) enable the computation of a retention rate. In this microsphere-based system, increased retention rates correspond to decreased iRBC deformability (Deplaine et al., 2011; Lavazec et al., 2012; Ndour et al., 2015) and are inversely correlated with EI as measured by ektacytometry. Microsphiltration has established that RBC infected by immature gametocytes are retained at high rates, similar to those of mature asexual stages, a finding confirmed by ektacytometry (Tiburcio et al., 2012). There is a deformability switch during gametocyte maturation from stage IV (rigid) to stage V (deformable), which is related to the expression of STEVOR parasite proteins (Sanyal et al., 2012). This phenotypic switch is not related to changes in the microtubule network in gametocyte cytoskeleton as assessed using the microtubule destabilizing agent trifluralin (Dearnley et al., 2016). In a multiethnic cohort in Benin, microsphiltration showed strong correlations between the deformability measured by microsphiltration and ektacytometry of circulating RBC, ethnicity (Fulani, Gando, Bariba, and Otamari) and infection status evaluated by rapid diagnostic test (Henry et al., 2018). In 2015, gametocyte retention in the microsphere filters was quantified through high-content imaging. This was the first pharmacological screening of potential gametocytes-stiffening molecules (Duez et al., 2015). Microsphiltration has recently been adapted to high-throughput screening purposes (Duez et al., 2018) where drug-induced stiffening induces rapid parasite clearance.

## Atomic Force Microscopy (AFM)

Observations using AFM (Binnig et al., 1986) are based on the depression of the cell surface with a specialized needle (cantilever) that scans the surface at constant velocity. In imaging mode, the cantilever tip scans the cell surface on the *x* and *y* axes. The high signal-to-noise ratio of AFM enables a 3D topological reconstruction of the sample surface with a nanometer-scale resolution. Lateral resolution is ∼1 nm and vertical resolution is 0.2 nm (Muller, 2008). In AFM force measurement, the cell is locally deformed by the sharp AFM cantilever tip on the *z* axis. The force produced is proportional to the deflection of a laser beam, producing a force-distance curve proportional to surface stiffness/elasticity of the membrane of the cell that is being deformed down to a piconewton resolution. With AFM the cell surface is depressed into the RBC unlike the micropipette suction that induces an outward extension of the RBC surface (Figure 1; Hochmuth, 2000). The key readout of AFM is the Young’s modulus E, where increased values mean an increased stiffness (Table 1). Pioneering AFM analyses used complex sample preparations (Aikawa et al., 1996; Nagao et al., 2000) that have been improved to a simpler method allowing the use of Giemsa staining (Li et al., 2006). AFM over the past decade has been largely automated and a widely used in cell mechanics, as reviewed recently (Yeow et al., 2017). AFM can analyze unfixed specimens and living cells in their buffer with minimal sample preparation. AFM confirmed the implication of TRPM7 and EBA-175 proteins in RBC invasion which were related to RBC deformability (Sisquella et al., 2017). Microsphiltration and ektacytometry have shown a low deformability of *P. falciparum* in the early stages of gametocytogenesis switching to an increased deformability at stage V gametocyte. AFM revealed that these changes result from a decrease in density of the parasite cytoskeleton network independent of microtubules (Dearnley et al., 2016). Recently, AFM coupled with infra-red spectroscopy, developed to go further in chemical analysis of cell membranes (Centrone, 2015), has resolved subcellular structure of asexual stages iRBC and thus could be useful to understand the mode of action of antimalarial drugs and their impact on deformability (Perez-Guaita et al., 2018).

## Optical Tweezers

Optical tweezers (or optical trapping) use a highly focused laser beam to generate a three-dimensional gradient of electromagnetic energy resulting in the trapping and controlling of microscopic objects. The force applied depends on the displacement of the beam waist. Two silica microbeads attached to diametrically opposed ends of an RBC are trapped by two laser beams and displaced to stretch the cell (Mills et al., 2004). Another variation of this method involves a single trap, with the cell attached to a glass plate and the trapped bead at the diametrically opposed end (Suresh et al., 2005). The set-up includes a photodiode to monitor the beam position and a microscope coupled to a camera to generate movies. Several computational approaches showed the relevancy of this method (Fedosov et al., 2011, 2014; Hosseini and Feng, 2012; Ye et al., 2013). Optical tweezers are more sensitive than micropipettes; micropipettes only give information about membrane elasticity whereas optical tweezers generate data on the mechanical behavior of the whole cell. In contrast to AFM, the stiffness and applied force of an optical trap can be changed instantaneously and flexibly by adjusting the intensity of the laser beam. Suresh et al. used optical tweezers to stretch and measure the elastic modulus of individual iRBC at different stages of infection. The shear modulus of iRBC was found to increase up to 10-fold on schizont-stage parasites compared to the healthy RBC (Suresh et al., 2005). These results were confirmed and associated with folding/unfolding duration of iRBC increasing also with parasite growth (Bambardekar et al., 2008). The involvement of Ring-infected Erythrocyte Surface Antigen (RESA) in deformability was assayed by using RESA gene-manipulated strains. RESA protein reduces deformability of host cells at the early ring stage of parasite development, but not at a more advanced stage with a marked effect at fever temperatures (Mills et al., 2007).

## Imaging Flow Cytometry

Imaging flow cytometry (IFM, Imagestream^®^) combines flow cytometry with microscopy. Ten to hundreds of thousands of single-cell pictures can be acquired in a matter of minutes including brightfield, scatter and fluorescent images. Hydrodynamically focused cells are trans-illuminated by a brightfield light source and orthogonally by laser(s). As a result, each cell image is broken-down into separate sub-images based on a range of spectral wavelengths. Semi-automatic analyses of RBC samples can identify discrete subpopulations based on morphological features. The morphology and size (i.e., projected surface area) of RBC are related to their ability to persist in circulation through their impact on the surface-to-volume ratio (Mohandas et al., 1980; Jaureguiberry et al., 2014; Pivkin et al., 2016). As an illustration, IFM has recently revealed that storage of RBC in blood bank conditions induces a spherocytic shift of some RBC due to a surface area loss (Roussel et al., 2017, 2018). These “storage-induced micro-spherocytes” are removed from the circulation (Roussel, 2019). A similar analysis of blood samples from three *Plasmodium knowlesi*-infected human subjects showed that sphericity was increased in iRBC compared with uninfected-RBC from the same patients (Barber et al., 2018). A reduction in the projected area of ring-infected RBC (*P. falciparum* culture *in vitro*) had also been observed using IFM and correlated with retention in microsphiltration (Safeukui et al., 2013).

## Discussion and Futures Perspectives

Early works demonstrated that iRBC deformability is an important determinant of malaria pathogenesis (Miller et al., 1971; Cranston et al., 1984). This notion was later reinforced by whole-blood approaches showing that RBC deformability and clinical severity of malaria attacks are correlated (Dondorp et al., 2002; Ishioka et al., 2016). This indirectly highlighted the major role of the spleen in malaria, partly through its ability to control the deformability of RBC. Interactions between uninfected RBC, infected RBC and the spleen are of importance in the pathogenesis of malaria and in the mechanisms of parasite clearance. Approaches exploring the alterations of RBC deformability induced by *P. falciparum* and the biophysical parameters regulating their bio-rheological behavior have significantly improved over the last decades. Historically, asexual mature stages (which are easy to purify *in vitro*), directly pathogenic through their ability to adhere to endothelial cells, were preferentially studied and were the target of most antimalarial drugs. It is now clear that the loss of RBC deformability begins soon after parasite invasion. Alteration of the biomechanical properties of infected and uninfected RBC potentially enhances their clearance by the spleen. RBC deformability-oriented approaches may therefore play a role in current and future strategies for malaria elimination (WHO, 2015). Enhanced stringency of splenic filtration also favors the mechanical trapping and clearance of the parasite, as sphericity and rigidity of RBC promote mechanical trapping and engulfment by macrophages (Sosale et al., 2015). Artemisinin derivatives, the currently recommended first-line antimalarial agents, display the major advantage of being active against both rings and mature stages, thus preventing sequestration in tissue capillaries. Unfortunately, artemisinin derivatives are threatened by the emergence of resistant parasites in South-East Asia (Dondorp et al., 2009). In this context, the need for new efficient antimalarials is pressing. Innovative methods have recently shown that a deformability-oriented approach enables the screening of transmission-blocking drugs (Duez et al., 2015, 2018). The promising spiroindolone family, which carries a potent antimalarial activity against asexual and sexual stages of *P. falciparum*, induces swelling of iRBC, impacting their deformability and inducing the fastest parasite clearance ever observed in humans (White et al., 2014).

This review of methods to study the deformability of RBC highlights the different information and outcomes they can each provide. Importantly, their combination enables a better understanding of deformability changes induced by parasite growth and/or by drugs. Confronting outputs from methods that challenge RBC deformability in qualitatively different ways (elongation, cylindrical squeezing, dumble-shape squeezing, membrane indentation, see Figure 1) opens the field of functional RBC morphology, with the aim of predicting the ability to transit through a microcapillary or a splenic slit. Further exploring RBC functional morphology in clinical situations should deepen our understanding of malaria pathogenesis with expected diagnostic, prognostic and therapeutic applications.

## Author Contributions

MD wrote the manuscript under the direction of PN and PB. BH, PN, and PB wrote and corrected the manuscript.

## Conflict of Interest

The authors declare that the research was conducted in the absence of any commercial or financial relationships that could be construed as a potential conflict of interest.
